# Modeling Change in Learning Strategies throughout Higher Education: A Multi-Indicator Latent Growth Perspective

**DOI:** 10.1371/journal.pone.0067854

**Published:** 2013-07-03

**Authors:** Liesje Coertjens, Vincent Donche, Sven De Maeyer, Gert Vanthournout, Peter Van Petegem

**Affiliations:** 1 Institute for Educational and Information Sciences, University of Antwerp, Antwerp, Belgium; 2 Centre for Statistics at the University of Antwerp (StatUA), University of Antwerp, Antwerp, Belgium; Chancellor College, University of Malawi, Malawi

## Abstract

The change in learning strategies during higher education is an important topic of research in the Student Approaches to Learning field. Although the studies on this topic are increasingly longitudinal, analyses have continued to rely primarily on traditional statistical methods. The present research is innovative in the way it uses a multi-indicator latent growth analysis in order to more accurately estimate the general and differential development in learning strategy scales. Moreover, the predictive strength of the latent growth models are estimated. The sample consists of one cohort of Flemish University College students, 245 of whom participated in the three measurement waves by filling out the processing and regulation strategies scales of the Inventory of Learning Styles – Short Versions. Independent-samples t-tests revealed that the longitudinal group is a non-random subset of students starting University College. For each scale, a multi-indicator latent growth model is estimated using Mplus 6.1. Results suggest that, on average, during higher education, students persisting in their studies in a non-delayed manner seem to shift towards high-quality learning and away from undirected and surface-oriented learning. Moreover, students from the longitudinal group are found to vary in their initial levels, while, unexpectedly, not in their change over time. Although the growth models fit the data well, significant residual variances in the latent factors remain.

## Introduction

How students go about their learning has been one of the core interests of educational researchers. This topic has been investigated with regard to many stages of formal education [1], [2], one important one being students in higher education. This issue has been looked at from different angles, with the Student Approaches to Learning (SAL) being a particularly long-lived one [3].

The SAL field is comprised of different theories describing students’ varying preferences for learning strategies [4]. Of the questionnaires associated with the SAL theories, Richardson [5] distinguishes the two most frequently used with campus-based students. Firstly, Biggs and colleagues’ Study Process Questionnaire (SPQ) [6] discerns two main ways to go about learning. The deep approach can be conceptualised as a combination of aiming for understanding and using strategies to create meaning; for example relating aspects of the content with one another. The surface approach can be understood as using memorizing techniques (learning by heart) with the aim of passing the course or task. Initially, a third approach was discerned, labelled achieving or strategic approach and capturing students’ strategies to maximise their grades. However, later studies revealed the validity of the two-factor structure [6]. Secondly, Entwistle and colleagues [7] developed the Approaches to Study Inventory (ASI) containing three orientations to learning. The meaning orientation and reproducing orientation can be conceptually linked to the deep and surface approach, respectively. The achieving orientation can be thought of in line with the achieving or strategic approach.

To these two questionnaires, Fox et al. [8] and Vanthournout et al. [9] add the Vermunt’s Inventory of Learning Styles Questionnaire (ILS), which maps four elements: (1) processing strategies; (2) regulation strategies; (3) conceptions of learning and; (4) learning orientations [10]. The first two elements –processing strategies and regulation strategies– are sometimes subsumed under the concept of learning strategies. These learning strategies can be related to the learning approaches or orientations described above. Processing strategies refer to those cognitive strategies that students apply whilst studying. In the ILS, two cognitive processing strategies map deep processing: critical processing and relating and structuring. Stepwise or surface processing is captured by the memorizing and analysing scale. Regulation strategies are those meta-cognitive activities students use to direct their learning process, such as planning or testing oneself. The ILS incorporates three such strategies. The self-regulation scale refers to directing the learning process oneself. External regulation captures the degree to which students seek guidance by the teacher or by the learning material. The lack of regulation scale expresses whether students are undirected in their learning, i.e. they do not steer themselves nor follow their teachers’ guidance.

The different theories on learning strategies assume linkage with academic performance. Concerning the SPQ, deep and surface processing strategies are expected to lead to higher or lower achievement, respectively [11]. A recent review study by Richardson and Bond [12] confirmed this: the correlation between grade point average (GPA) and deep and surface approaches is small positive and small negative, respectively. Using the ASI or an adaptation of it (RASI or ASSIST), a number of studies found small positive correlations between meaning orientation and grade [13], [14], [15], though the study by Provost and Bond [16] did not detect an association. The reproducing orientation was in most studies found to have a small negative association with achievement [13], [15], [16].

Regarding the ILS, empirical research shows positive and weak-to-moderate correlations between deep processing and academic achievement [17], [18], [19], [20]. For surface processing, the memorizing strategy is unrelated to performance whilst the evidence for the analysing strategy is mixed between a positive association [17], [18] and absence of a correlation [19], [20]. For the regulation strategies, the evidence on self-regulation and external relation is unequivocal whilst the unregulated learning strategy is repeatedly found to be related to lower academic achievement [18], [19], [20].

Though learning strategies are clearly not the sole predictor of academic achievement in general, for the three learning strategy questionnaires (SPQ, ASI and ILS), deep- or meaning oriented learning has a small positive correlation with academic achievement. For surface/reproducing learning, in most studies, a small negative association with academic achievement is found. Last, unregulated learning is associated with lower academic performance.

The association between learning strategies and learning outcomes is not only important during higher education but also afterwards, for example in the working context. It is presumed that deep learning during higher education is linked to being a reflective and adaptive practitioner, who participates in lifelong learning [21], [22]. Though more research is clearly needed to test this presumption, Hoeksema and colleagues [23] detected that deep learning correlates with career success whilst surface learning was found to hamper it. Self-directed learning was also found to be positively associated with the amount of work-related learning [24].

Next to literature on the link between learning strategies, achievement and lifelong learning, numerous studies in the SAL field debate whether learning strategies should be conceptualised as a trait or a state [3], [25]. Though some view them to be fixed personality-like characteristics [26], there is a body of literature on the influence of person-related factors, such as age and motivation (for a review, see [27]), supporting the view of learning strategies as a state. Next to this, the influence of contextual factors - for example, elements in the learning environment such as teachers’ approaches on students’ learning strategies or assessment - has been described [27], [28], [29]. Moreover, a number of studies have detected change in learning strategies over time [22], [30], [31].

### Change in Learning Strategies

Change in learning strategies over time has been on the one hand assessed using cross-sectional designs. Relying on the SPQ, both Gow & Kember [32] and Biggs [33] found that students in higher years at university scored lower on both the deep and achieving approach. No significant differences between the different years was noted for the surface approach. Using the Revised ASI (RASI), Richardson [34] detected lower levels for the deep approach in higher years, while they were higher for the surface approach. There was no significant difference between the years concerning the strategic approach.

It can be argued that found effects in cross-sectional designs could alternatively be explained by varying group composition between the years. Therefore, researchers have assessed changes in learning strategies by using repeated measurements of for example the SPQ, ASI or ILS with the same students [22], [35], [36]. Historically, this research has relied on pre-test post-test designs over a short interval of time [8], [37], [38], [39], [40]. Recently, more than two measurement waves have been taken into account and longer time intervals have been allowed for [36], [41], which comply with the criteria for longitudinal research as put forward by Singer and Willet [42]. For each of the three theoretical frameworks, we will briefly discuss the findings of these longitudinal studies.

Relying on Biggs and colleagues’ framework, Phan [30], Gordon and Debus [41] and Jackling [43] noted an increase in deep processing over time. On the other hand, Zeegers [36] concluded that it remained constant. These results clearly contradict the findings in the cross-sectional studies mentioned earlier, in which the deep approach was found to be lower for students in later years of higher education. Concerning surface processing, Gordon and Debus [41] detected a decreasing reliance, while Jackling [43] and Zeegers [36] found a constant trend. The change in the achieving approach was only investigated by Gordon and Debus [41], who concluded that it remained constant over time. Using the ASSIST [44], Reid and colleagues [22] noted a decrease in both the deep and the strategic approach over the first year of medical training, while the surface approach did not alter. Over the second year, the surface and deep approach remained stable, while the strategic approach continued to decrease.

Five longitudinal studies use the Vermunt framework and the ILS to map changes throughout higher education [31], [35], [45], [46], [47]. Concerning meaning-directed learning [45] or deep processing strategies [35], [46], [47] an increase was found. However, Vanthournout [31] concluded that only the relating and structuring scale increases, while the degree of critical processing remains constant. Stepwise processing and its subscale analysing was found to remain constant over time [31], [45], [46], [47], while the degree of memorizing decreased over time [46] or showed a quadratic trend with a rise after an initial decrease [31]. Concerning the regulation strategies, self-regulation was found to increase [31], [46], [47], or to remain constant [35]. External regulation, on the other hand, decreased [31], [46], [47]. Lastly, undirected learning was found to remain constant [35], [45], [47] or decrease over time [31], [46].

### Restraints of the Statistical Techniques used to Assess Change in Learning Strategies

An examination of how longitudinal studies within the SAL field are undertaken statistically reveals a strong reliance on comparisons of manifest scale scores over time. Per student, the scores on the items for each scale are averaged at each wave. Subsequently, repeated measures (M)ANOVA are relied upon to compare the mean factor scores over time [35], [43]. This type of analysis discerns whether students, *on average,* increase or decrease in terms of a particular learning strategy scale.

When average growth is estimated by comparing manifest scale scores over time, two important elements are overlooked. First, it remains veiled whether students follow a comparable growth trajectory or whether they differ in their growth over time. Studies on this differential change in learning strategies are explicitly called for in literature [48]. However, evidence concerning the differential growth in learning strategies during higher education is scarce. At present, only two studies have looked at this in an in-depth fashion [30], [31].

Second, all studies on the changes in learning strategies over time have relied upon manifest scale scores to draw conclusions with regard to latent factors (e.g. deep learning decreases during higher education). Hereby, a number of measurement issues are overlooked. By using manifest scale scores, the measurement error associated with learning strategy items, the ordinal nature of Likert scale items and the assumption of measurement invariance are ignored. In what follows, we will first detail the prior findings concerning differential growth. Next, the limitations of manifest scale scores are discussed.

### Differential Change in Learning Strategies

A number of studies have investigated the influence of initial learning strategies over a short period of time [27]. At the course-level, Wilson and Fowler [49] detected that students scoring high on the deep approach did not vary in their reliance on this strategy between a conventional and action learning course. One the other hand, students judged ‘typically surface’ reported greater use of deep learning strategies in the action learning course. Studies by Gijbels and colleagues [50] and Vanthournout [31] confirm that initial learning strategies influence the change in these strategies during a course.

Four studies have looked at the differential evolution in learning strategies over longer periods of time [30], [31], [46], [48]. Two studies performed preliminary analysis on subgroups of students. Donche et al. [46] examined whether students’ changes in learning strategy scales during their time in higher education was dependent on the learning profile upon entry into higher education. Relying on cluster analysis and subsequently paired-samples t-tests per cluster, the authors concluded that there is some evidence that subgroups of students develop in different ways. Nienemin and colleagues [48] detected small differences in the development of learning strategies among students scoring below average, compared to their peers scoring above average.

The two other studies have used more advanced analysis to model differential evolution explicitly. Relying on a multilevel model, Vanthournout [41] found a differential evolution in change over time for the critical processing, self-regulation, analysing and external regulation scales. For the last two scales, this correlated negatively with students’ initial level: students scoring higher at the start of higher education tended to decrease their reliance, while those scoring lower initially tended to increase it. Using a latent growth model, Phan [30] concluded upon a comparable differential growth in deep processing: students scoring lower on deep processing at the start of their undergraduate program were found to increase their reliance more rapidly.

### Limitations of Manifest Scale Scores

Up to present, all studies on the change in learning strategies over time have used manifest scale scores. These manifest scale scores ignore however three measurement issues: measurement error, the ordinal nature of Likert scale items and measurement invariance over time. By relying on multi-indicator latent growth (MILG) analysis, average as well as differential growth can be estimated while taking each of these measurement issues adequately into account.

First, as confirmatory factor analyses on learning strategy questionnaires confirm, items do not perfectly measure a certain concept, but have measurement error [17]. Such measurement error can be explicitly modelled in MILG analysis [51], thereby estimating a latent mean per measurement wave, which can be subsequently compared over time [52], [53].

Second, the three measurement instruments described (SPQ, ASI & ILS) use Likert scales. By averaging the scores on the items for one learning strategy scale and applying a repeated-measures (M)ANOVA, it is implicitly assumed that the manifest scale scores are continuous. Yet, whether this assumption holds true, is debatable for Likert scales [52], [54]. In MILG analysis, the ordinal nature of these scales can again be explicitly accounted for, allowing us to ‘… make inferences about change on an interval metric when all we have are data on ordinal metric’ ([52], p. 304).

Third, the comparison of learning strategy factor scores over time is based upon the assumption of measurement invariance [55], [56]. What the learning strategy questionnaire actually measures needs to be equivalent at each wave to allow for comparisons over time [57]. This assumption applies to (M)ANOVA’s, multilevel models and MILG analysis alike. (M)ANOVA’s and multilevel models do not provide the opportunity to falsify this assumption, given that they rely on the manifest scale scores. MILG analysis on the other hand models growth in the latent scale scores. Together with the growth trend, the factor structure (factor loadings and item difficulty) is thus estimated. This allows verification of whether factor loadings and item difficulty remains constant over time (i.e. measurement invariance).

Furthermore, MILG analysis allows for testing how good a growth trajectory predicts the true, latent change in a learning strategy scale. Not only fit indices, but also R^2^ parameters, indicate the predictive power of the growth trajectory [58]. Moreover, residual variances suggest whether additional predictors are needed to adequately estimate the change in learning strategy scales over time.

### The Current Research

The current research further analyses the data of the Donche et al. [46] study, in which repeated measures ANOVA were used. By accounting for measurement error, measurement variance and the ordinal nature of the data, we aim to answer the following research questions: how do student’s processing and regulation strategies develop on average? (Research Question 1, RQ1), is there differential growth in learning strategies? (RQ2) and, how much variance in the latent factors is explained by the growth factors? (RQ3).

## Materials and Methods

### Ethics Statement

For research in higher education, ethics approval and written consent is not required by Belgian law. The Law on Experiments on Humans (7^th^ May 2004) obliges ethics approval and consent for an experiment, whereby ‘experiment’ is defined as “each study or research in which human persons are involved with the goal of developing appropriate knowledge for the performance of health professions” (“elke op de menselijke persoon uitgevoerde proef, studie of onderzoek, met het oog op de ontwikkeling van de kennis eigen aan de uitoefening van de gezondheidszorgberoepen”, 2004050732/N, Article 2, paragraph 11). The current research is not related to the performance of health professions and is therefore implicitly exempt from ethics approval and written consent. We underline that participation was at each wave on a voluntary basis and that the students, who were all adults, could stop their participation at any moment. There was no penalty for students who chose not to participate, nor were they rewarded for participation with, for example, student counselling regarding learning strategies. Confidentiality of the results was guaranteed by the research team.

### Measurement

Learning strategies are investigated by focusing on two malleable components of the learning patterns of the Vermunt framework: cognitive processing and regulation activities [59]. The scales used in this study stem from the ‘Inventory of Learning Styles – Short Version’ (ILS-SV), which has been validated for first-year Flemish University College students [60]. Processing strategies can be viewed as the cognitive activities a student applies whilst studying. In the ILS-SV, four scales for cognitive processing strategies are distinguished: memorizing, analysing, critical processing and relating and structuring. The first two are related to stepwise processing while the last two map deep processing. Regulation strategies are metacognitive activities that students undertake. To map regulation strategies, the ILS-SV discerns three scales: external regulation, self-regulation and lack of regulation. For all seven scales, the items are scored ranging from (1) ‘I never or hardly ever do this’ to (5) ‘I (almost) always do this’. For each scale, Table 1 provides the number of items, an example item (translated from Dutch) and the estimate of reliability. Due to the sensitivity of Cronbach alpha to the number of items [61], [62], the mean inter-item correlation is a more appropriate measure of reliability for scales with few items [63]. At each wave, all scales – each containing either 4 or 5 items – fall within the.2 to.5 range for good reliability (see Table 1).

**Table 1:** Learning strategy scales of the ILS-SV questionnaire, number of items, item examples (translated from Dutch) and range of scale reliability.

Scales	Items	Item example	Mean inter-item correlation
*Processing strategies*			
*Stepwise/surface*			
Memorizing	4	I learn definitions by heart and as literally as possible.	.34–.39
Analysing	4	I study each course book chapter point by point and look into each piece separately.	.33–.36
*Deep*			
Critical processing	4	I try to understand the interpretations of experts in a critical way.	.32–.39
Relating and structuring	4	I compare conclusions from different teaching modules with each other.	.35–.46
*Regulation strategies*			
External regulation	5	I study according to the instructions given in the course material.	.20–.27
Self-regulation	4	I use other sources to complement study materials.	.28–.35
Lack of regulation	4	I confirm that I find it difficult to establish whether or not I have sufficiently mastered the course material.	.31–.38

### Design, Respondents and Data Availability

The research took place in a Flemish University College in which one cohort of students was followed. In March of the first academic year (from September to June), all first-year students participated in the research during scheduled lecture slots. The same cohort was questioned again in May of the second and the third year. Each wave provided adequate response rates, as shown in Table 2. Of this cohort, 254 students participated in the three waves of data collection. 245 of those provided complete data on all ILS-SV items at each of the three waves. They constitute the longitudinal group for which growth is assessed in this study. The data are freely available upon request to the first author.

**Table 2:** Response rate per measurement wave.

	Wave 1	Wave 2	Wave 3
Number of registered students	1412	731	561
Number of respondents	1047	515	392
Response rate (%)	74.1	70.4	65.8
Number of respondents with complete data	1037	507	363
Participants with complete data at each wave (longitudinal group)	245	245	245

As is common in longitudinal studies, not all students participated in the three waves of data collection. Firstly, some students stopped their studies or did not pass the exams, which is quite common in the first year of University College in Flanders (e.g. of the cohort under study, only 51.7% of the first year students were enrolled in the second year). For the first wave, independent-samples t-tests undertaken on the seven learning strategy scales (see Table 1), indicated that students from the longitudinal group (N = 245) scored significantly higher on the analysing scale, but scored lower on the lack of regulation scale than their peers who participated in the first wave, but did not participate on all three occasions (N = 802; *t*(1045) = −3.217, *p*<.01, Cohen’s *d* = .23 and *t*(1045) = 5.55, *p*<.001, Cohen’s *d* = .40 respectively). For the second wave, comparable significant differences in the same two scales were noted (N = 270, *t*(513) = −2.7, *p*<.01, Cohen’s *d* = .24 and *t*(510.39) = 5.25, *p*<.001, Cohen’s *d* = .46 respectively). Although all effects are small [64], the results suggest that students who persist in their studies through the third year do not constitute a random subset of students entering University College. This is in line with findings on the link between learning strategies and academic achievement (see Introduction). Therefore, for the analysing and lack of regulation scale, findings on the longitudinal group can only be generalised to the sub-population of students persisting in University College.

Secondly, some students persisted in their studies, but not in the research. Comparing students of the longitudinal group (N = 245) to their peers answering at the third wave but not participating three times (N = 124), reveals that the former score higher on the self-regulation strategy scale, though the effect is also small (*t*(367) = −2.057, *p*<.05, Cohen’s *d* = .23). This result warrants caution in generalizing the findings for this scale to the subgroup of students persisting in University College.

### Multi-indicator Latent Growth Model

**Figure 1:**
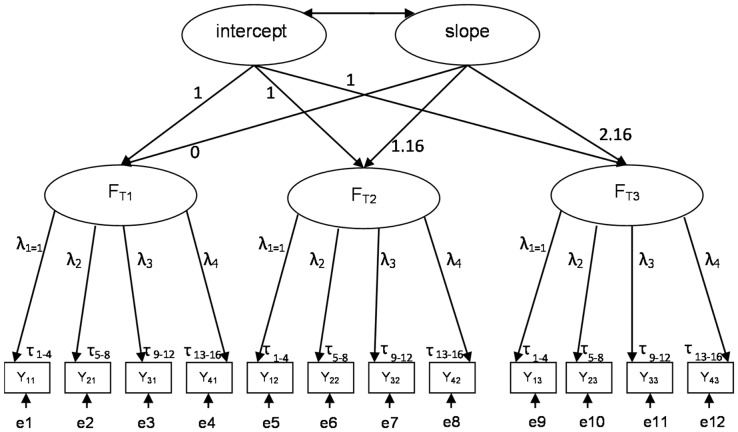
Multi-indicator latent growth model.

To assess change in a learning strategy scale, we opted for a multi-indicator latent growth model (MILG) [54]. Fig. 1 exemplifies such a model, which consists of two levels. The first level accounts for the common variation in the multiple indicators. A factor (e.g. the latent concept memorizing) is measured at three moments, using the same four items each time (Y_1_–Y_4_). An individual’s score on an item at a certain point in time (Y_ijt_ e.g. Y_i31_ represents the score for individual i on the third item at the first wave) is predicted by a latent factor (e.g. F_T1_). The second-order factors – intercept and slope – serve to explain the mean and covariance structure of these latent factors [47]. The intercept parameter signifies the average initial value for the scale (in our case: the value in March of the first year of University College), while the slope parameter estimates whether, on average, there is a significant increase or decrease in the scale scores per unit of time (in this case, 12 months). For more technical detail on latent growth modelling, we refer to Duncan et al. [65] and Voelkle et al. [58]. Since the data consist of three waves, constant and linear models can be identified [52], [53], [54]. Due to data gathering at unequal time intervals (14 months between wave 1 and 2 and 12 months between wave 2 and 3), the values of the factor loadings for the slope have been adjusted to 0, 1.16 (being 14/12^th^) and 2.16, respectively [51], [54].

Besides the estimates for average growth (intercept and slope), the latent growth analysis also details the differences between students [65]. A significant intercept variance indicates that students differ in their initial values on the scale, while slope variance suggests that students vary in their growth trajectory. If both variances prove significant, the covariance is informative as well [51].

Next to this, the output of the MILG model details the explained variance in the latent factors. The R^2^ is provided per latent factor, indicating which percentage of the variance in this latent factor is explained by the combination of the intercept and slope. Next to this, the MILG model output provides, for each latent factor, an estimate of the residual variance, indicating how much variance in this latent factor is left unexplained [54].

As mentioned above, making inferences about change in a learning strategy scale based upon repeated measurement, hinges upon the assumption of measurement invariance [35], [36], [39]. Longitudinal measurement invariance indicates that the definition of the latent construct is comparable over time [57]. In Fig. 1, the two elements of this measurement invariance are depicted. Factor loadings are constrained equal over time (e.g. λ_2_), indicating that one increase in a factor (F) represents the same increase in Y at different waves [51]. Next to this, the thresholds are presumed equal as well. With five-point Likert scales, there are four thresholds per item (τ_1–4_, see Fig. 1). Threshold invariance implies that the percentage of students’ choosing a Likert point should be comparable across waves [52].

Prior research has investigated this issue of longitudinal measurement invariance for the longitudinal sample under consideration [66]. Results confirmed complete longitudinal measurement invariance for five learning strategy scales (memorizing, critical processing, relating and structuring, self-regulation and lack of regulation). With regard to the external regulation and the analysing scale, one and two thresholds, respectively, failed to reveal equivalence over measurement moments. These small measurement inequivalences are modelled in the partial measurement invariance models and taken into account when modelling growth for the external regulation and analysing scale.

Multi-indicator latent growth analyses were performed using Mplus 6.1. Due to the data’s ordinal nature, the use of the conventional maximum likelihood (ML) estimation procedure could not be justified [52]. The distribution free estimation procedure weighted least squares means-variance (WLSMV) was, therefore, employed [54], [67]. Due to this WLSMV, the change in Chi^2^ and degrees of freedom cannot be calculated in a straightforward fashion. “The difference in chi-square values for two nested models using the […] WLSMV chi-square values is not distributed as chi-square” ([56], p. 501). Therefore, a scaling correction (DIFFTEST function) is relied upon [68], of which only the p-value should be interpreted.

Though more simulation studies are required to establish the sample size requirements in the case of MILG models with ordinal data, research suggests the requirements do not differ compared to ML estimation. Moreover, the WLSMV was found to perform well with small samples, at least as well as the ML [69]. Sample sizes of 200 or greater resulted in accurate estimates [70] on the condition that variables were not too skewed [71]. The skewness of the ILS-SV items in this sample ranged between 0.012 and 1.134, and, following DiStefano and Hess [72], indicated no reason for concern. Therefore, the sample of 245 students is adequate to estimate the MILG model.

In assessing the fit of each latent growth model, a series of fit indices was relied upon. However, as investigated by De Roche [73], the cut-off values of these indices are not independent of the number of waves and respondents. In the case of three time points and 250 respondents, De Roche [73] suggests examining the Chi^2^, CFI, NNFI/TLI and RMSEA. However, the performance of the first may diminish with non-continuous data. Therefore, the CFI, NNFI/TLI and RMSEA are considered key. The first two proved robust in terms of sample size and number of waves, allowing the cut-off of.95 to be maintained. With regard to the latter, an adjusted cut-off of.08 is suggested [73]. Furthermore, we followed Wu et al.’s [53] suggestion and examined the change in the CFI and RMSEA. If, from the (complete or partial) measurement invariance model to the linear growth model, the CFI or the RMSEA deteriorates, this suggests that adding the growth factors did not help explain the patterns observed in the data [53].

## Results

**Figure 2:**
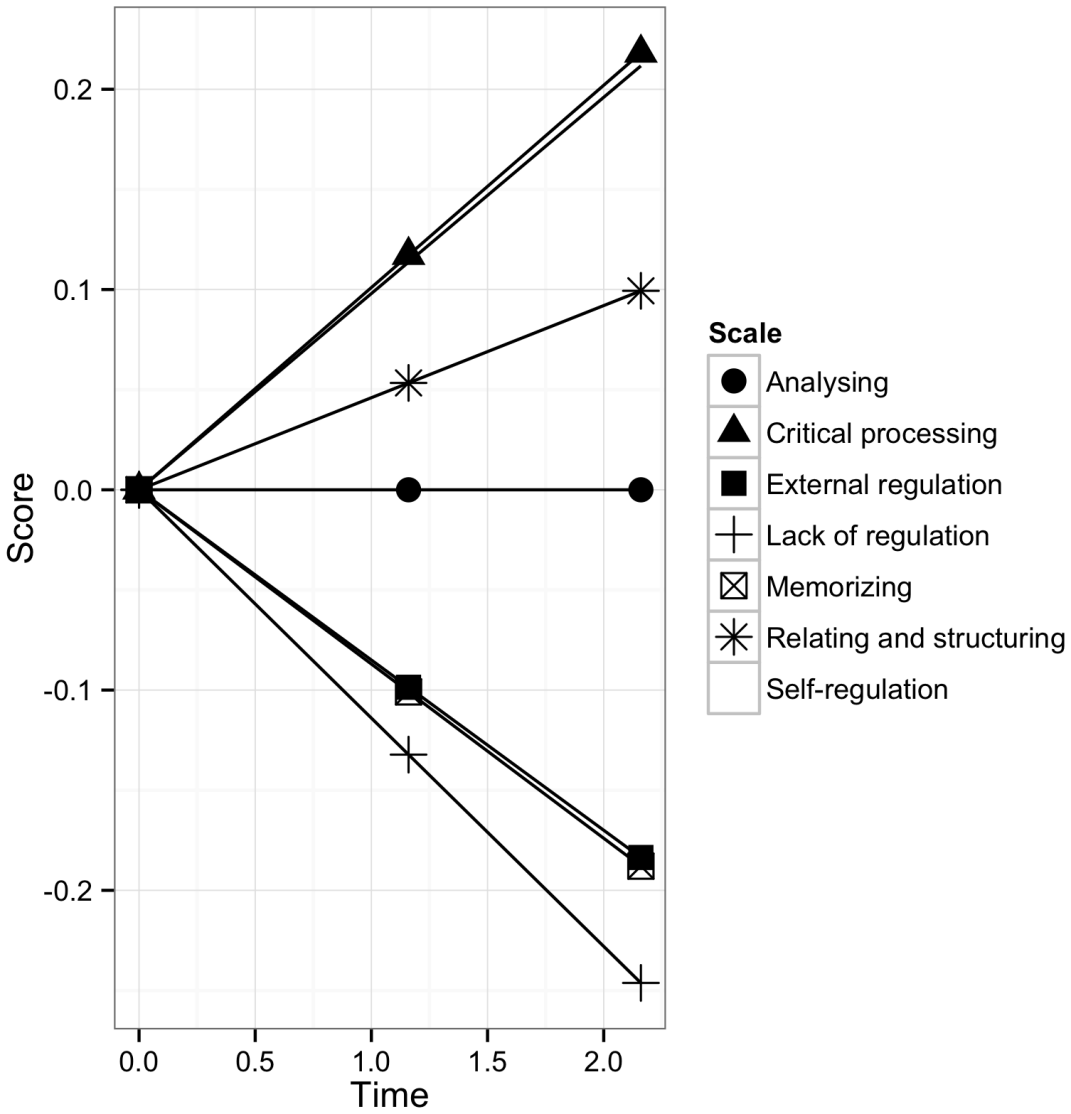
Average growth trajectories for the processing and regulation scales.

For each learning strategy scale, the fit of the MILG model is provided in Table 3. Table 4 shows the explained and residual variance at each wave for the seven scales whilst Table 5 presents the parameter estimates. The average growth trend for each scale is also displayed in Fig. 2.

**Table 3:** Fit indices for measurement invariance and latent growth models.

		?^2^	df	p	CFI	NNFI/TLI	RMSEA
							(90% conf. interval)
Memorizing	invariant measurement model	66.127	67		1.000	1.001°	.000–.037
	linear model	64.888	68		1.000	1.002°	.000–.034
Analysing	partial invariant measurement model	105.520	65	**	.969	.968	.032–.068
	linear model	106.209	66	***	.969	.969	.031–.067
Critical processing	invariant measurement model	82.600	67		.989	.989	.000–.051
	linear model	87.010	70		.988	.989	.000–.051
Relating and structuring	invariant measurement model	94.712	67	*	.985	.986	.019–.058
	linear model	109.679	70	*	.979	.980	.030–.065
External regulation	partial invariant measurement model	158.145	107	**	.953	.954	.029–.058
	linear model	159.781	110	**	.954	.956	.027–.057
Self-regulation	invariant measurement model	67.851	65		.998	.998	.000–.041
	linear model	66.983	66		.999	.999	.000–.039
Lack of regulation	invariant measurement model	103.456	67	*	.978	.979	.028–.064
	linear model	101.928	70	*	.981	.982	.023–.061

*** *p*<.001;

** *p*<.01;

* *p*<.05; ° the NNFI/TLI can fall out of the 0–1 range when the df is larger than the χ^2^ without implying an erroneous or just-identified model [73].

**Table 4:** R^2^ and residual variances (Res var) at the three waves.

	Wave 1		Wave 2		Wave 3	
	R^2^	Res var	R^2^	Res var	R^2^	Res var
Memorizing	.658	.198*	.652	.205***	.684	.189*
Analysing	.626	.191**	.793	.091*	.852	.076
Critical processing	.689	.128**	.679	.134**	.642	.159***
Relating and structuring	.671	.135**	.714	.11***	.67	.135***
External regulation	.593	.17***	.637	.142**	.598	.167***
Self-regulation	.506	.244**	.658	.193***	.929	.039
Lack of regulation	.669	.106***	.905	.022	.674	.104***

Note. All values of R^2^ were significant at the.01 level;

*** *p*<.001;

** *p*<.01;

* *p*<.05; The R^2^ and residual variance are not standardized and therefore do not add up to one.

**Table 5:** Parameter estimates for the multi-indicator latent growth models°.

	Slope			VAR Intercept			VAR Slope		COV	
	Est.	SE	p	Est.	SE	p	Est.	SE	Est.	SE
Memorizing	−.087	.027	**	.381	.097	***	.010	.037	−.004	.051
Analysing	.003	.025		.319	.074	***	.027	.028	−.003	.036
Critical processing	.101	.022	***	.284	.047	***	put to zero		not estimated	
Relating and structuring	.046	.022	*	.274	.039	***	put to zero		not estimated	
External regulation	−.085	.024	***	.248	.041	***	put to zero		not estimated	
Self-regulation	.098	.028	***	.250	.087	**	.017	.034	.042	.042
Lack of regulation	−.114	.021	***	.215	.038	***	put to zero		not estimated	

*** *p*<.001;

** *p*<.01;

* *p*<.05; ° Due to the MILG model, for each scale, the parameter estimate for the intercept is zero.

### Processing Strategies

For the memorizing scale, results indicate good fit for the linear growth model (Table 3). Moreover, compared to the invariant measurement model, fit did not deteriorate. At the different time points, the linear growth model succeeded in explaining between 65% and 68% of the variance in the latent factor. Nevertheless, as shown in Table 4, residual variance ranging between 18% and 20% remained at the three waves.

**Figure 3:**
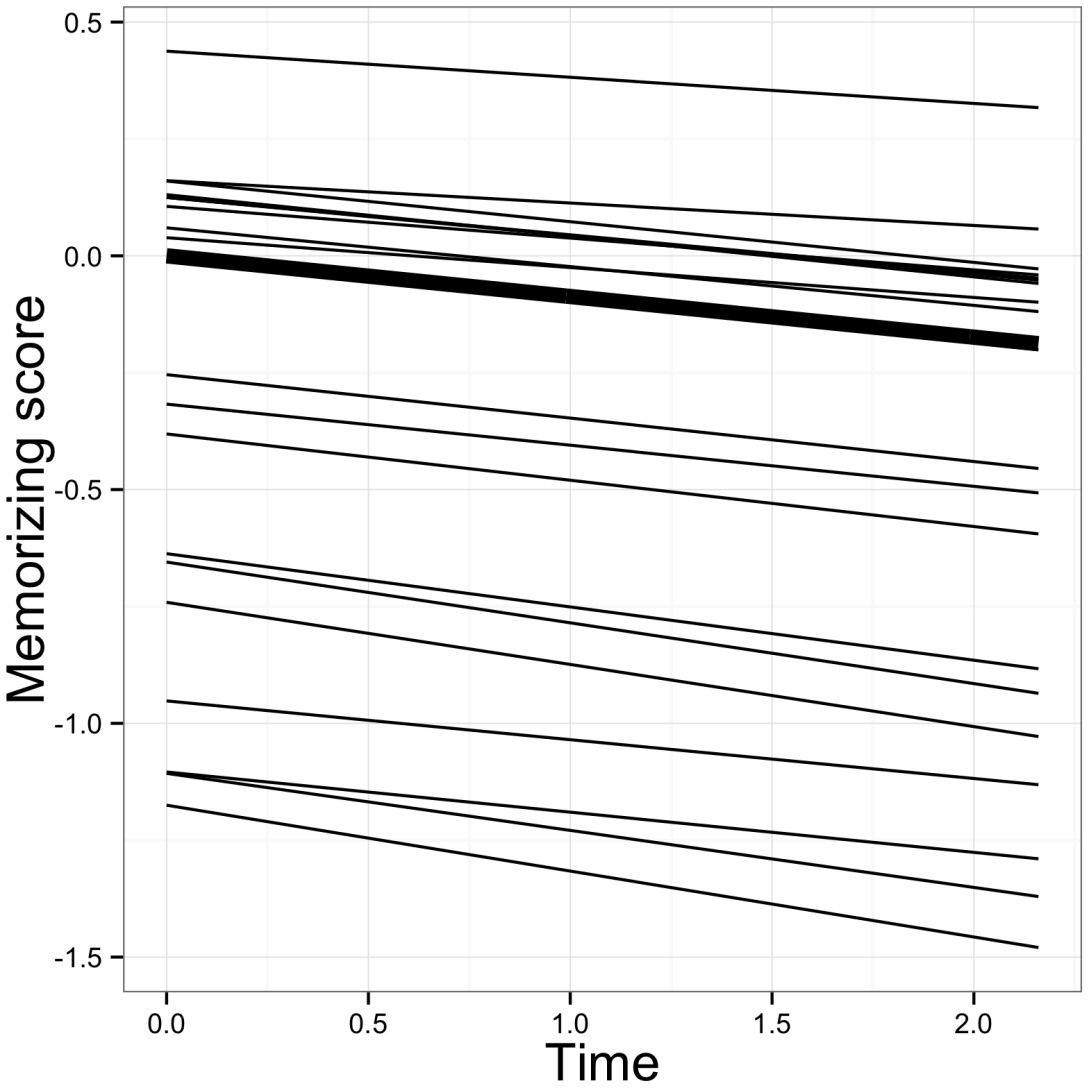
Average and predicted individual growth trajectories for the memorizing scale.

The parameter estimates of the growth model are given in Table 5. For memorizing, a linearly decreasing reliance on the strategy during time in higher education is noted (*Est. slope* = −.087, *se* = .027, *p*<.01). This trend is depicted in Fig. 2. The variance for the intercept proved significant (*Est VAR intercept* = .381, *se* = .097, *p*<.001, see Table 5), meaning that there are significant differences in students’ initial levels of memorizing. Fig. 3 shows the average growth trajectory as well as the predicted individual growth trajectory for a random subset of 20 students. It shows that there are differences at the start of higher education and that the general trajectory is not neatly followed by all students. The null hypothesis for the slope variance was, however, not rejected (*Est VAR slope* = .010, *se* = .037, *p*>.05).

**Figure 4:**
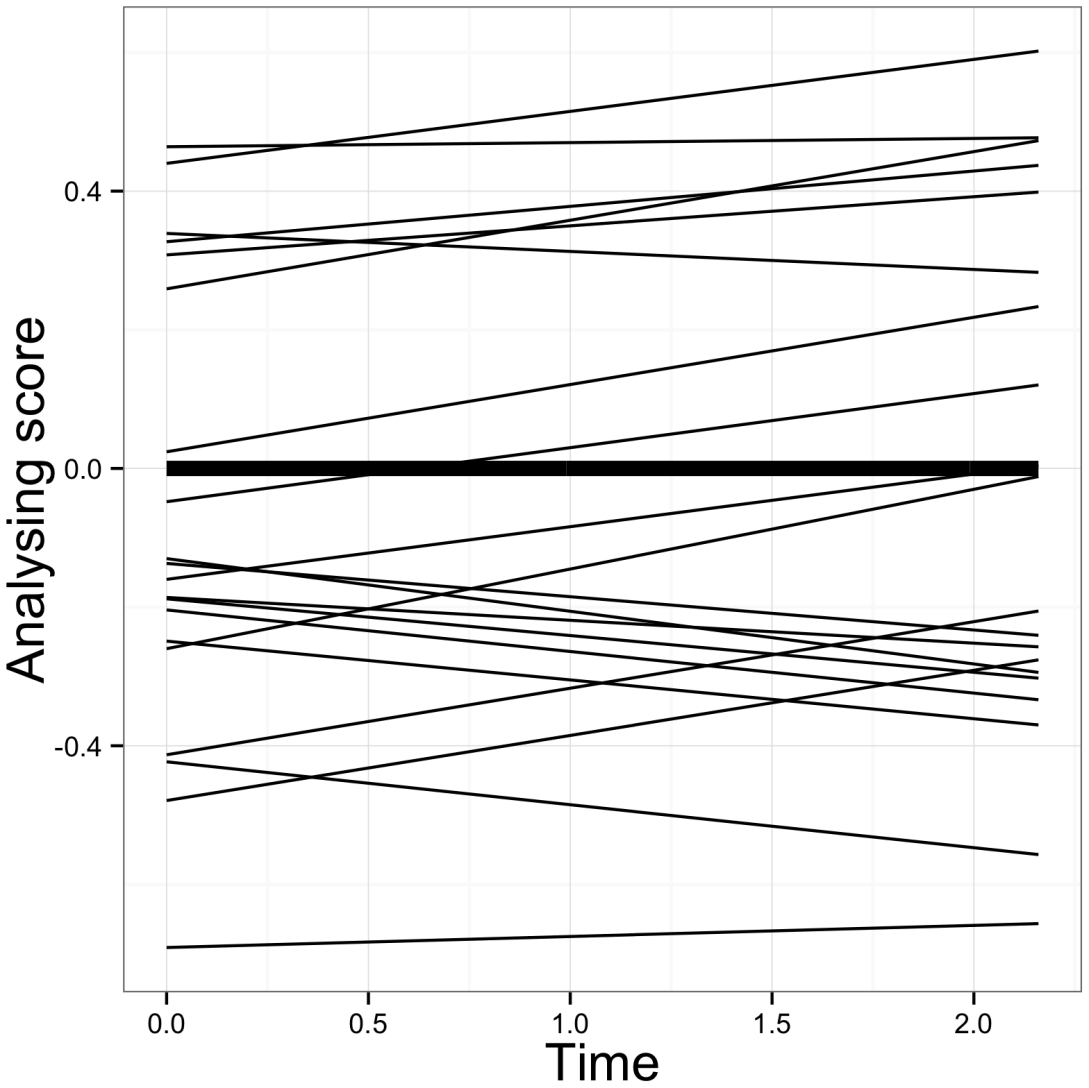
Average and predicted individual growth trajectories for the analysing scale.

As far as the analysing scale is concerned, indices suggested good model fit and remained at the same level compared to the partial invariant measurement model (see Table 3). The portion of the variance explained by the growth parameters increased from 62% to 85% over the waves, leaving significant residual variances only at the first two waves (see Table 4). As Fig. 2 shows, the degree of analysing remains constant over time. Fig. 4 shows for a random subset of 20 students the predicted individual growth trajectory in analysing next to average growth trajectory. The variance parameter for the slope did not prove significant, while students are estimated to vary in their initial level of analysing (see Table 5).

For the first of the deep processing scales, critical processing, results indicated a negative but non-significant variance for the slope (*Est. slope* = −.030, *se* = .027, *p*>.05). Following the suggestion by Muthén [74], the variance for the slope was constrained to zero. Subsequently, the model showed good fit, also compared to the invariant measurement model (see Table 3). Parameter estimates suggest a linear increase in critical processing over time (see Fig. 2). Concerning the differential growth, there was again significant intercept variance (see Table 5).

The results for the relating and structuring scale indicated a correlation between the intercept and the slope larger than one. To resolve this, the non-significant variance for the slope was constrained to zero. Successively, good model fit was found and the indices remained close to the level of the previous model (see Table 3). While significant residual variances were noted for the three latent factors, the model explains between 67% and 71% of the variance in latent factors scores (see Table 4). At the start of the study, students varied significantly in the degree to which they used relating and structuring. An increase in relating and structuring over time is noted as well (see Table 5 and Fig. 2).

### Regulation Strategies

For the external regulation scale, the latent variable covariance matrix was not positive definite either, due to a negative, though non-significant, slope variance (*Est. VAR slope* = −.031, *se* = .028, *p>*.05). After putting this variance to zero, the fit of the latent growth model was adequate, and comparable to the partial invariant measurement model (see Table 3). The linear growth model explains between 59% and 63% of the variance in latent factor scores over time. Nevertheless, there is significant residual variance at each wave (see Table 4). Students are found to vary in their initial level of external regulation, and are noted to decrease their reliance on this regulation strategy over the course of their time in higher education (see Table 5 and Fig. 2).

**Figure 5:**
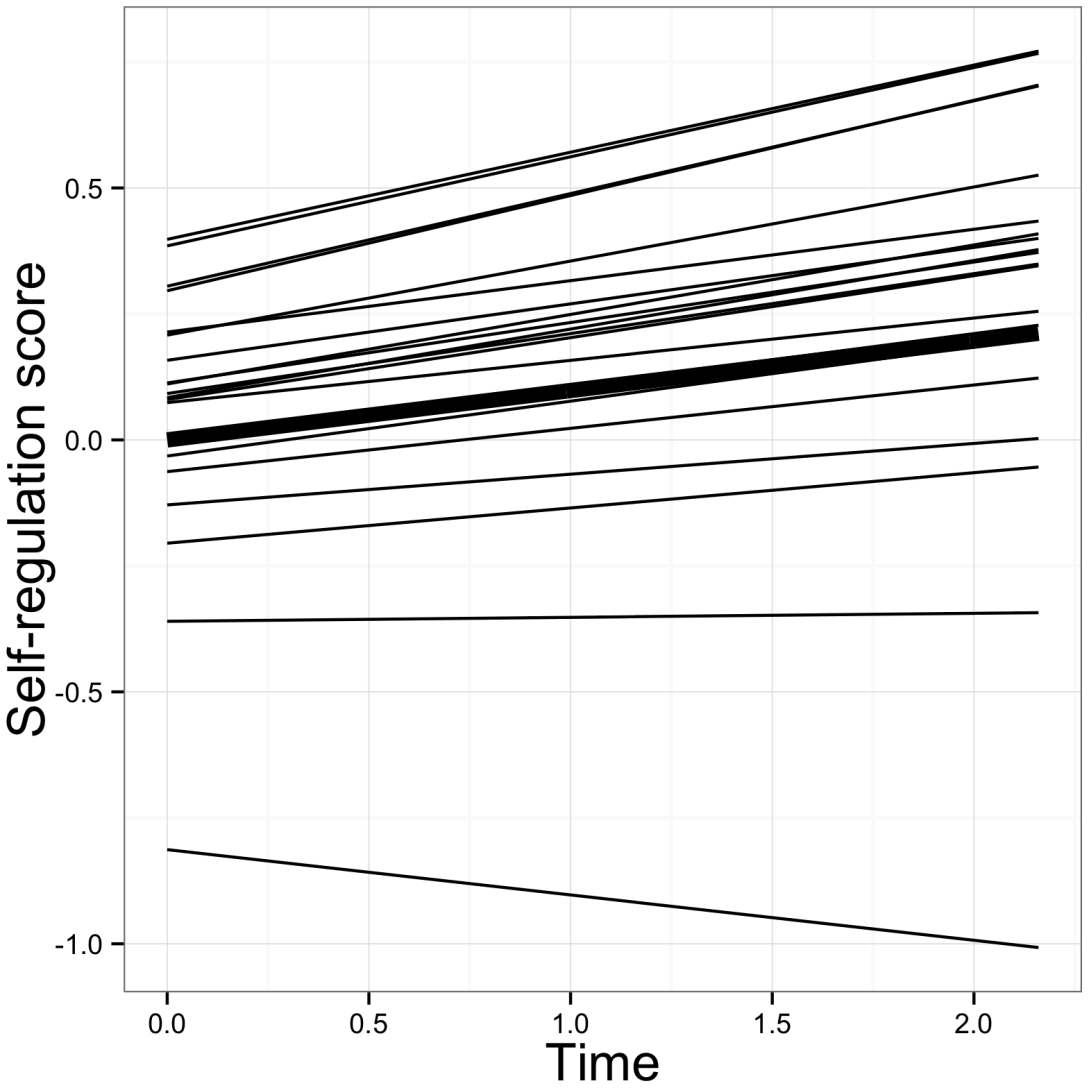
Average and predicted individual growth trajectories for the self-regulation scale.

As far as the self-regulation scale is concerned, excellent fit was shown by the indices (see Table 3). Over the three waves, the R^2^ improved from 50% to 93% (see Table 4). As shown in Fig. 2, self-regulation increases over the course of this study. For 20 students, the individual predicted growth trajectory is shown in Fig. 5. Examining the estimates for the variance parameters (see Table 5), a difference in students’ initial levels of self-regulation is noted, while the general increasing trend can be assumed to be valid for all students.

Lastly, the lack of regulation scale again showed a negative, though not significant, estimate for the slope variance (*Est. VAR slope* = −.012, *se* = .018, *p*>.05). Constraining this variance to zero provided a good model fit, which was better compared to the fit for the invariant measurement model. Results suggest that students vary significantly in their initial score on this scale and decrease their lack of regulation during higher education (see Table 5 and Fig. 2). The explained variance per latent factor ranges from 67% to 91%, leaving a significant residual variance of 10% at the first and at the third wave (see Table 4).

## Discussion

Research on student learning has increasingly focussed on investigating the change in learning strategies during time spent in higher education. However, this domain relies predominantly on traditional statistical techniques, such as repeated measures ANOVA. The present research is innovative in the way that it investigates the average and differential growth trajectory in a more accurate and thorough fashion, through the use of multi-indicator latent growth analysis.

The results regarding the average growth trajectories in processing and regulation strategies (RQ1) are in line with Donche et al.’s [46] findings. Using the MILG model, results indicate that critical processing, relating and structuring, as well as self-regulation, increased over time. Memorizing, external regulation, and lack of regulation were found to decrease whilst the degree of analysing remained constant. However, for both the analysing and lack of regulation scales, the students persisting in their studies in a non-delayed manner form a biased follow-up sample. Therefore, the findings for these scales can only be generalized to the subgroup of students persisting in University College. For the self-regulation scale, significant differences were found between students participating in the third wave and those belonging to the longitudinal group. Taking this attrition into account, students following a non-delayed educational career in University College generally do seem to develop in the direction of more deep learning, moving away from surface-oriented and unregulated learning. Students persisting in the research as well seem to increase their self-regulation over time.

This finding of change in learning strategies over time adds to the trait-state debate. When taking measurement error, the ordinal nature of the Likert scale data and the partial measurement invariance adequately into account, results indicate variability in students’ learning strategies over time. Thus, in line with prior longitudinal research [22], [30], [31], [41], [45], [47], the proposition of learning strategies as stable characteristics over time is refuted [75], even within an educational context that is relatively stable (i.e. the same University College).

Findings on differential growth (RQ2) indicate significant intercept variance for all scales, which is in line with prior research [30], [31]. This finding suggests that in the longitudinal sample consisting of students persisting in their studies in a non-delayed manner and providing complete data at each wave (N = 245), students vary in their initial degree of processing and regulation.

Next, the results suggest an absence of significant variability in slopes for all scales, contradicting prior preliminary analysis of the data, which relied upon discerning subgroups [46]. The results are also at odds with studies in which advanced statistical analysis was used [30], [31]. A first explanation could be a lack of statistical power required to reject the null hypothesis. Perhaps, there is differential growth, but given the sample size (N = 245) significance could not be reached. A second explanation could be the selectivity of the longitudinal sample. Contrary to Vanthournout [31] but in line with Phan [30], only students providing complete data at each wave were retained in the present research. This choice stems from a limitation of MILG analysis, which does not allow for missing data at the item level.

To gauge the effect of attrition on the conclusions, the data were analysed in an alternative way. In contrast to the MILG analysis as presented, all students who participated at least once (N = 1182) were included by relying on manifest scale scores and by modeling growth using a maximum likelihood procedure. Results revealed that for four scales the conclusion of absence of slope variance remained (memorizing, relating and structuring, analysing and lack of regulation). For the critical processing scale and the external regulation scale, the slope variance was constrained to zero. For the self-regulation scale, however, the slope variance did just reach significance (*Est. VAR slope* = .047, *se* = .023, *p*<.05; *Est. covariance* = −.012, *se* = .030, *p*>.05). Thus, only for the self-regulation scale conclusions differed when considering attrition. Though not in line with prior findings [30], [31], for all other scales the evolution over time in learning strategies appears genuinely comparable. Further methodological research should focus on allowing missingness at the item level so that the strengths of the MILG can be combined with allowing for missing data. More research into the differential growth in learning strategies is warranted as well.

Concerning the explained and residual variance (RQ3), results indicate that between 50.6% and 92.9% of the variance in latent factors of processing and regulation strategy scales was accounted for by the linear growth trajectory. At the same time, for each of the learning strategy scales, at least two of the three errors variances proved significant. Though powerful predictors, the growth factors thus appear insufficient to predict the varying levels of the latent factors. On the one hand, these results plead for the use of nonlinear models in future research and, on the other hand, they warrant inclusion of other predictors to explain students’ changes in learning strategies over time [53], such as prior education, gender or motivation to study.

Since the strength of any research study lies in the recognition of its limitations, two important constraints should be considered. The first is the issue of attrition, which is common to longitudinal studies, especially when longer time intervals are involved [47], [76]. A second constraint concerns the number of measurement waves. With three data points, constant or linear growth trajectories can be estimated [52], [53]. However, more complex functions could clarify the issue of residual variances. Moreover, they are also of interest from a remedial point of view. Vermetten et al. [38], for example, suggested the need to investigate the developmental pattern in a more fine-grained fashion. ‘Is it a one-way, gradual process in which students become more self-regulated, deep-level learners? Or is it a capricious pattern, with periods of stability followed by periods of change?’ (p. 238) Thus, to better trace the actual development and detect opportunities for stimulating learning strategy development, future research should preferably span over longer periods of time [77] and include more than three measurement points over time [78].

The constraints of the present study notwithstanding, results for the multi-indicator latent growth analyses confirm the presence of a developmental trend in learning strategies during higher education towards high-quality learning. Students are, however, found to vary only in their initial levels of processing and regulation, but not in their development in these learning strategies during their time in higher education.
